# Antenatal magnesium sulphate administration for fetal neuroprotection: a French national survey

**DOI:** 10.1186/s12884-017-1489-z

**Published:** 2017-09-13

**Authors:** Clément Chollat, Lise Le Doussal, Gaëlle de la Villéon, Delphine Provost, Stéphane Marret

**Affiliations:** 1 Assistance Publique - Hôpitaux de Paris, Service de Médecine et Réanimation Néonatales de Port-Royal, Groupe Hospitalier Cochin, Broca, Hôtel-Dieu, Hôpital Port-Royal 123 bd de Port-Royal, 75014 Paris, France; 2 INSERM U1245, Team “Genetics and Pathophysiology of Neurodevelopmental Disorders”, Rouen University, Normandie University, Faculty of Medicine, 22 Boulevard Gambetta, 76183 Rouen Cedex, France; 3 Department of Neonatal Paediatrics and Intensive Care – Neuropaediatrics, Rouen University Hospital, Hôpital Charles Nicolle 1 rue de Germont, 76000 Rouen, France; 4 Department of Anaesthetics and Intensive Care, Rouen University Hospital, Hôpital Charles Nicolle 1 rue de Germont, 76000 Rouen, France

**Keywords:** Magnesium sulphate, Neuroprotection, Neonatology, Very preterm infants, National survey

## Abstract

**Background:**

Magnesium sulphate (MgSO_4_) is the only treatment approved for fetal neuroprotection. No information on its use is available in the absence of a national registry of neonatal practices. The objective of our study was to evaluate the use of MgSO_4_ for fetal neuroprotection in French tertiary maternity hospitals (FTMH).

**Methods:**

Online and phone survey of all FTMH between August 2014 and May 2015. A participation was expected from one senior obstetrician, one senior anaesthetist and one senior neonatologist from each FTMH. Information was obtained from 63/63 (100%) FTMH and 138/189 (73%) physicians. Use of MgSO_4_ for fetal neuroprotection, regimen and injection protocols, reasons for non-use were the main outcome measures.

**Results:**

60.3% of FTMH used MgSO_4_ for fetal neuroprotection. No significant difference was observed between university and non-university hospitals or according to the annual number of births. Protocols differed especially in terms of the maximum gestational age (3% <28 WG, 71% <33 WG, 18% <34 WG and 8% < 35 WG). Eighty seven percent of centers using MgSO_4_ prescribed retreatment when necessary, but according to non-consensual modalities in terms of number of treatments or between-treatment intervals. Injections and monitoring were mostly performed in the delivery room (97%) but also in the recovery room in one half of hospitals. Lack of experience (52%), absence of a written protocol (49%) and national guidelines (46%) were the reasons most commonly reported to explain non-use of MgSO_4_ as a neuroprotective agent.

**Conclusions:**

Sixty percent of FTMH used MgSO_4_ for fetal neuroprotection, but according to heterogeneous regimens. National guidelines could allow standardization of practices and better MgSO_4_ coverage.

## Background

Protection of the immature brain of premature infants constitutes a crucial challenge for obstetricians and neonatologists. Although the survival of premature infants is continuously improving [1], their neurological outcome remains a major concern, as preterm birth is associated with neurodevelopmental impairments such as neuromotor deficits, cognitive deficits, learning disabilities, behavioral and psychiatric disorders and neurosensory deficiencies [2]. The prevalence of cerebral palsy (CP) in Europe slowly decreased from 1.90 to 1.77 per 1000 live births between 1980 to 2003, but still remains high [3]. Almost 40% of individuals with CP were born preterm and the risk of CP increases with decreasing gestational age [4]. Neuroprotection in the context of preterm birth is the subject of extensive research, but few strategies have currently been demonstrated to be effective. There is strong evidence to support antenatal magnesium sulphate (MgSO_4_) infusion in order to prevent CP in context of prematurity [5]. Based on animal and human observational studies that demonstrated a neuroprotective effect of MgSO_4_ [6, 7], five randomised controlled trials (RCTs) published between 2002 and 2008 were conducted to evaluate its effect [8–13]. Four meta-analysis of these five trials demonstrated that antenatal MgSO_4_ treatment significantly reduced the risk of CP in very preterm 2-year-old infants (for the Cochrane meta-analysis: overall RR 0.68; 95%CI 0.54 to 0.87; five trials, 6145 infants) [5, 14–17]. Sixty-three women had to be treated in order to prevent CP in one child (95% CI 43 to 155). However, no statistically significant effect on infantile mortality was observed [5]. In the light of these convincing results, several national authorities (USA, Australia and New Zealand, Canada, UK, Belgium and Ireland) have recommended antenatal administration of MgSO_4_ in women at imminent risk of very preterm birth in order to prevent cerebral palsy [18–24].

In France, no guidelines have been drafted in view of the ongoing debate concerning this evidence. No information on the use of MgSO_4_ in France is available in the absence of a national registry of neonatal practices.

## Methods

### Study design

This study was designed to evaluate the use of MgSO_4_ prior to preterm birth in all French tertiary maternity hospitals (FTMH) to describe the various protocols used and to analyze the reasons for non-use. This questionnaire-based study was conducted between August 2014 and May 2015 among all FTMH. Sixty-three FTMH were identified (61 in metropolitan France and two in overseas territories). In France, MgSO_4_ is usually prescribe by obstetricians. Midwives or nurses administer it under the responsibility of the anaesthetists, in case of side effects. Paediatricians, on the other hand, are most often initiators of the MgSO_4_ protocol at maternity. The point of view of theses three medical specialists is therefore crucial to adress this issue in a holistic way. Accordingly, in each center, the Heads of Departments of Obstetrics, Anaesthesia and Neonatology were contacted by mail and/or phone. Surveys were completed by the Head of Department or a senior physician. Each participant was able to answer the survey online, by e-mail or by phone. The Local Ethics Committee of Rouen University Hospital ruled that no formal ethics approval was required for such a current practice survey. Informed consent to participate was obtained from all participants involved in this study. The survey was designed and conducted using Google Forms software (https://www.google.com, Mountain View, CA, USA).

### Collected data

The first part of the questionnaire comprised three general questions:

1. In your opinion, what are the advantages of administering MgSO_4_ to the mother before preterm birth?
2. Have you ever read one or more scientific articles on this topic?
3. Do you use MgSO_4_ in your center for neuroprotection prior to very preterm birth?

The second part of the questionnaire focused on MgSO_4_ users. Participants answered 20 questions about their MgSO_4_ administration protocol and their patterns of use.

The third part concerned MgSO_4_ non-users. Participants had to indicate their reasons for not using MgSO_4_:

- lack of knowledge on the subject
- lack of scientific evidence
- expected benefit/risk balance not in favor of the use of MgSO_4_
- lack of local experience
- no written protocol in the department
- reluctance of obstetricians or anaesthetists or paediatricians or all three.

### Data analysis

Data were recorded on an Excel database. Results were analyzed descriptively. Statistical analysis was performed with Statview 5.0 (SAS Institute Inc.). Statistical analysis was done with the chi-squared test. A value of *p* < 0.05 was considered statistically significant.

## Results

A total of 138 responses were obtained from the 189 physicians contacted (73%). A response to the survey was obtained from all 63 FTMH (100%) and from 54% of anaesthetists (34/63), 76% of obstetricians (48/63) and 89% of paediatricians (56/63). Respondents were distributed as follows: 40% (56/138) from paediatricians, 35% (48/138) from obstetricians and 25% (34/138) from anaesthetists. Respondents were distributed equally between university hospitals and non-university hospitals and according to the annual number of births per center. Among the respondents, 91% were familiar with the neuroprotective effect of MgSO_4_ and 79% had read at least one scientific article on this topic, with no significant difference between specialities (Table 1). Thirty-eight (60%) of the 63 centers used MgSO_4_ prior to preterm birth in order to protect the infant’s immature brain. No significant difference was observed according to the type of hospital (university hospital or non-university hospital, *P* = .47) or the annual number of births in each hospital (<3000 births per year or >3000 births per year, *P* = .71). Among the FTMH that routinely used MgSO_4_, 95% had a written administration protocol per hospital. The maximum gestational age for MgSO_4_ administration ranged between 32 (27/38 centers, 71%) and 33 WG (7/38 centers, 18%), while three centers continued to administer MgSO_4_ up to 34 WG. However, one center did not prescribe MgSO_4_ after 28 WG. The majority of centers (35/38, 92%) prescribed a loading dose of 4 g followed by maintenance treatment at a dose of 1 g per hour for 12 h. Thirty-three FTMH repeated the regimen when necessary. The modalities of subsequent courses were less clearly defined: 50% of written protocols did not specify the minimum interval between two doses and 76% did not specify the maximum number of doses. Nevertheless, the subsequent course was started within 24 h for 26% of centers, between 24 and 48 h for 16% of centers, and after 48 h for 8% of centers. Seven FTMH (18%) repeated the infusion once, one center repeated the infusion twice, and one center did not set any limits. Calcium channel blockers were coprescribed by 33 centers (87%) (Table 2). Contraindications and criteria for discontinuation of the MgSO_4_ infusion are shown in Table 3. MgSO_4_ was administered in the delivery room (97%), but also in the recovery room (50%), intensive care unit (24%) or obstetrics unit (21%). The reasons for non-use of MgSO_4_ are described in Table 4. None of the anaesthetists who did not use MgSO_4_ answered the question of non-use of MgSO_4_ (0/4). Lack of experience (52%), lack of a written protocol (49%) and the absence of national guidelines (46%) were the reasons most commonly reported by respondents to explain the absence of use of MgSO_4_ as a neuroprotective agent in their unit.

**Table 1:** Description of respondents

	Speciality					Center n/N (%)
	Anaesthetists n/N (%)	Obstetricians n/N (%)	Paediatricians n/N (%)	Total n/N (%)	*P*	
Number of respondents	34/63 (54)	48/63 (76)	56/63 (89)	138/138 (100)	<.001	63/63 (100)
University Hospital	25/41 (61)	32/41 (78)	36/41 (88)	93/123 (76)	.66	41/63 (65)
Non-university Hospital	9/22 (41)	16/22 (73)	20/22 (91)	45/66 (68)		22/63 (35)
Annual number of births						
<3000	17/36 (47)	23/36 (64)	33/36 (92)	73/108 (68)	.49	36/63 (57)
>3000	17/27 (63)	25/27 (93)	23/27 (85)	65/81 (80)		27/63 (43)
Familiar with the neuroprotective value of MgSO_4_	30/34 (88)	44/48 (92)	51/56 (91)	125/138 (91)	.76	N/A
Had read at least one article on this topic	24/34 (71)	40/48 (83)	45/56 (80)	109/138 (79)	.36	N/A

Values are n/N (%)

Percentages may not sum to 100 because of rounding

**Table 2:** MgSO_4_ protocols

MgSO_4_ indications	n/N (%)
Pre-eclampsia	46/63 (73)
Neuroprotection	38/63 (60)
Pre-eclampsia and neuroprotection	37/63 (59)
None	17/63 (27)
Written protocol for indication and administration	36/38 (95)
Maximum gestational age	
34	3/38 (8)
33	7/38 (18)
32	27/38 (71)
31	0/38 (0)
30	0/38 (0)
29	0/38 (0)
28	1/38 (3)
MgSO_4_ regimen	
Loading dose of 4 g then maintenance with 1 g/h for 12 h	35/38 (92)
Loading dose of 4 g then maintenance with 2 g/h for 12 h	1/38 (3)
Only maintenance with 1 g/h for 12 h	2/38 (5)
Repeat MgSO_4_ if birth does not occur after the first course of MgSO_4_	33/38 (87)
Minimum interval between 2 treatments	
< 24 h	10/38 (26)
24 to 48 h	6/38 (16)
> 48 h	3/38 (8)
Not specified by protocol	19/38 (50)
Maximum number of treatments	
2	7/38(18)
3	1/38 (3)
No maximum number	1/38 (3)
Not specified by the protocol	29/38 (76)
Start of MgSO_4_ administration in the case of imminent delivery < 4 h (without delaying obstetric management)	34/38 (90)
Coprescription of calcium channel blockers	33/38 (87)

Values are n/N (%)

Percentages may not sum to 100 because of rounding

**Table 3:** Contraindications, criteria for discontinuation of the infusion and place of administration and monitoring

Contraindications	n/N (%)
Renal failure	33/38 (87)
Electrolyte disorders	19/38 (50)
Cardiovascular disease	27/38 (71)
Respiratory distress	2/38 (5)
Myasthenia gravis	23/38 (61)
Delivery <30 min	18/38 (47)
Digitalis interaction	22/38 (58)
MgSO4 intolerance	1/38 (3)
Stop the infusion if	
Oliguria <120 mL/4 h	2/38 (5)
Oliguria <100 mL/4 h	29/38 (76)
Hemodynamic instability	31/38 (82)
Respiratory rate < 12/min	6/38 (16)
Respiratory rate < 10/min	28/38 (74)
Hyporeflexia	35/38 (92)
Disorders of consciousness	33/38 (87)
Other	4/38 (1)
Place of administration	
Delivery room	37/38 (97)
Recovery room	19/38 (50)
Intensive care unit	9/38 (24)
Obstetrics unit	8/38 (21)

Values are n/N (%)

Multiple answers were possible

**Table 4:** Reasons why MgSO_4_ was not used

	Speciality					Center n/N (%)
	Anaesthetists n/N (%)	Obstetricians n/N (%)	Paediatricians n/N (%)	Total n/N (%)	*P* ^a^	
Lack of knowledge	0/4	2/12 (17)	4/21 (19)	6/37 (16)	.95	6/25 (24)
Lack of scientific evidence	0/4	2/12 (17)	2/21 (10)	4/37 (11)	.55	3/25 (12)
Lack of guidelines	0/4	4/12 (33)	11/21 (52)	15/37 (41)	.29	13/25 (52)
Benefit-risk balance	0/4	2/12 (17)	3/21 (14)	5/37 (14)	.85	5/25 (20)
Lack of experience	0/4	7/12 (58)	10/21 (48)	17/37 (46)	.55	17/25 (68)
Lack of written protocol	0/4	7/12 (58)	9/21 (43)	16/37 (43)	.39	13/25 (52)
Reluctance of obstetricians	0/4	2/12 (17)	2/21 (10)	4/37 (11)	.55	3/25 (12)
Reluctance of anesthesiologists	0/4	2/12 (17)	3/21 (14)	5/37 (14)	.85	5/25 (20)
Reluctance of pediatricians	0/4	2/12 (17)	2/21 (10)	4/37 (11)	.55	4/25 (16)

Values are n/N (%)

Multiple answers were possible

^a^Comparison between the answers provided by obstetricians and pediatricians, as anesthesiologists did not answer this question when they did not use MgSO_4_

## Discussion

### Main findings

This study provides an update on the reported use of MgSO_4_ for fetal neuroprotection in France, in the absence of a national registry of neonatal practices in France. Our survey showed that 60% (38/63) of FTMH stated that they used MgSO_4_ for fetal neuroprotection. This practice is not widespread and remains heterogeneous in France, despite a good knowledge by respondents of the scientific evidence and the benefits of MgSO_4_ in this context.

Another important finding is that the absence of national guidelines is one of reasons for non use MgSO_4_ for fetal neuroprotection.

### Strenghts and limitations

This study presents a number of limitations: 1) our findings are based on physicians’ statements and not on written protocols; 2) only one representative of each speciality in each hospital was asked to complete the survey, which may not be representative of the whole team; 3) although at least one response was obtained from each FTMH, the various specialities were not equally represented (anaesthetists: 54%, obstetricians: 76% and paediatricians: 89%, *P* < .001); 4) albeit we have a clear idea of the number of tertiary maternity units that use MgSO4, actual coverage remains unknown as data for each center were not available (i.e number of preterm birth and number of fetuses exposed to MgSO_4_). We took into account only the reported policies and not the actual use of MgSO_4_.

Despite these major limitations, this survey is useful to describe the conditions of use of MgSO_4_ in real-life practice and to understand the lack of generalisation of such a protocol in France.

### Interpretation

Our findings cannot be compared with others because there are no previous surveys about the use of MgSO_4_ for fetal neuroprotection. Our study highlighted the diversity of regimens and protocols in France. According to international guidelines, the most consensual regimen consisted of a 4 g loading dose following by 1 g/h maintenance treatment. However, heterogeneous practices were observed for several procedures, particularly the possibility of retreatment and the minimum interval between two treatments, as these particular issues are not clearly discussed in the scientific literature (especially in the five randomised controlled trials and meta-analysis). Meta-analysis of the individual data of participants in these five RCT is currently underway and could clarify these issues [25]. In our study, only one center (3%) prescribed MgSO_4_ up until 28 WG, whereas 71% of centers prescribed MgSO_4_ up until 32 WG. This difference could be explained by the number of women who need to be treated to avoid one case of cerebral palsy: 29 before 28 WG and 63 before 32 WG [5]. No data are available in the literature concerning the indication for MgSO_4_ after a gestational age of 32 WG, but a randomised controlled trial is ongoing (MAGENTA study protocol) [26]. However, in France, 18% and 8% of MgSO_4_ users centers prescribed this agent up until 33 WG and 34 WG, respectively. A similar heterogeneity is also observed in international guidelines. For example, the Australia and New Zealand Binational Clinical Practice guidelines established 30 WG as a limit [27]. Canadian, Belgian and Irish guidelines recommend that MgSO_4_ administration be proposed before 32 WG [21, 23, 24]. UK guidelines proposed the use of MgSO_4_ up until 30 WG and indicated that it should be considered up until 34 WG [22]. The American College of Obstetricians and Gynecologists recommended the development of specific guidelines, especially concerning gestational age, “in accordance with one of the larger trials” but with no specific details [18].

Contraindications and criteria for discontinuation of the infusion seem to be more consensual in our survey, with minor differences for oliguria or bradypnea cut-off values. Almost all units administered MgSO_4_ in the delivery room, while one half of units administered MgSO_4_ also in the recovery room. Administration of this treatment obviously requires close cardiorespiratory monitoring and the place of administration should be determined according to the unit’s usual practice and the possibility of close monitoring by appropriate staff (nurse, midwife and/or anaesthetist). Drafting a written protocol seems essential to define the treatment regimen and to optimise its implementation. A few studies have demonstrated the feasibility of this type of protocol, with adequate selection of women at high risk of imminent preterm birth, ensuring optimal MgSO_4_ coverage for infants born preterm with a minimum of unnecessary maternal exposure [28–31]. The American College of Obstetricians and Gynecologists published a patient safety checklist, which could be used to improve targeting and safety of MgSO_4_ administration [32].

Non-users reported lack of experience and the absence of a written protocol to explain their practice. Only a few respondents reported a low level of scientific evidence (4/37, 11%, representing 3/25 maternity units, 12%), suggesting that the majority of respondents consider that MgSO_4_ is beneficial for fetal neuroprotection. In contrast, the absence of national guidelines was reported by 46% of non-users, representing 13/25 maternity units, 52%. In France, national guidelines are necessary to ensure more widespread use of MgSO_4_. At the present time, MgSO_4_ is the only specific fetal neuroprotective medical treatment able to improve the neurodevelopment of preterm infants. As the development of preterm children is a long process which can be influenced by several environmental factors, optimal brain neuroprotective strategies comprising pharmacological interventions associated with educational measures need to be determined.

## Conclusions

Sixty percent of FTMH used MgSO_4_ for fetal neuroprotection. Administration protocols differed from one center to another, particularly concerning the maximum gestational age, the possibility of retreatment, and the place of administration and monitoring. National guidelines could allow standardization of practices and better MgSO_4_ coverage. In the future, the use of MgSO_4_ should be as systematic as that of corticosteroids, allowing paediatricians to rightfully ask obstetricians: “Have you injected both corticosteroids and MgSO_4_?” in case of preterm birth.

## Abbreviations

CP: Cerebral palsy
FTMH: French tertiary maternity hospitals
MgSO_4_: Magnesium sulphate
